# Supplementary material for: Fermented Chinese Herbs Improve the Growth and Immunity of Growing Pigs through Regulating Colon Microbiota and Metabolites
Source: Animals (Basel). 2023 Dec 15;13(24):3867. doi: 10.3390/ani13243867 (PMC10740985; doi:10.3390/ani13243867)
Supplement: Supplementary file 1 [file animals-13-03867-s001.zip › Supplementary Figures and Table S1 caption.pdf]

Supplementary Materials

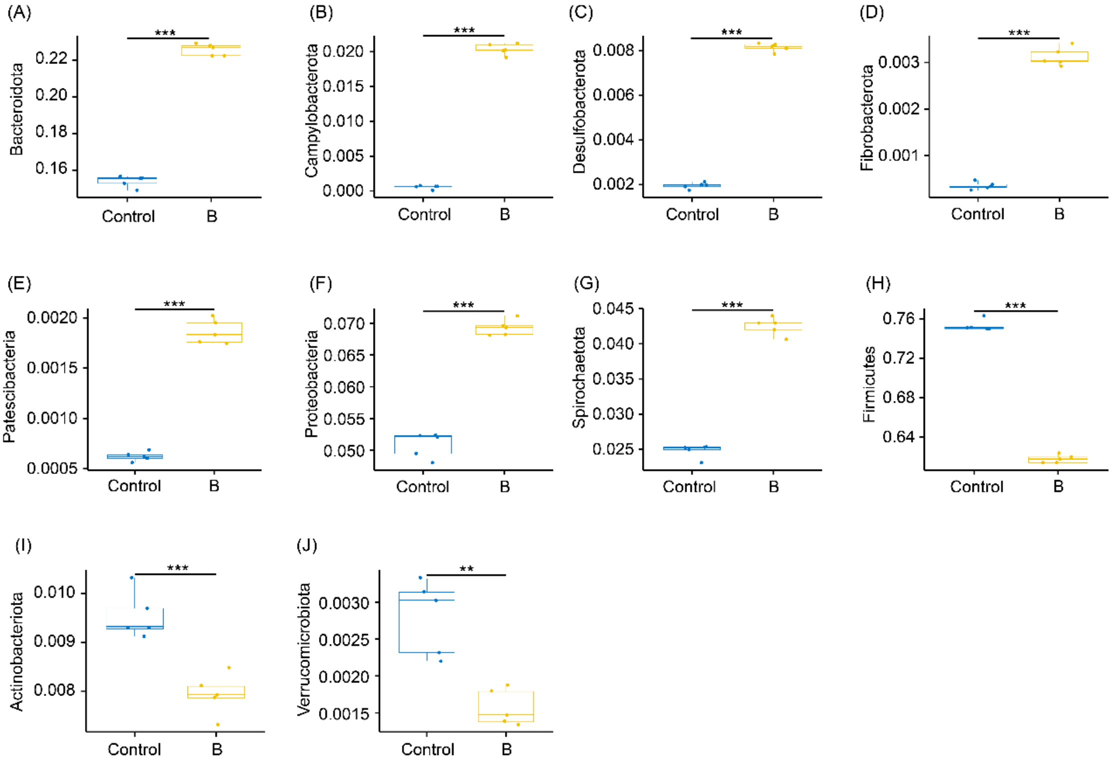

Figure S1. Relative abundances of dominant phyla in the colonic microbiota of growing pigs. (**A**) Bacteroidota; (**B**) Campylobacterota; (**C**) Desulfobacterota; (**D**) Fibrobacterota; (**E**) Patescibacteria; (**F**) Proteobacteria; (**G**) Spirochaetota; (**H**) Firmicutes; (**I**) Actinobacteriota; (**J**) Verrucomicrobiota. Pigs in the control group were fed basal diet, and the diet of group B was the basal diet with 2% FCHs. ** $p < 0.01$; *** $p < 0.001$.

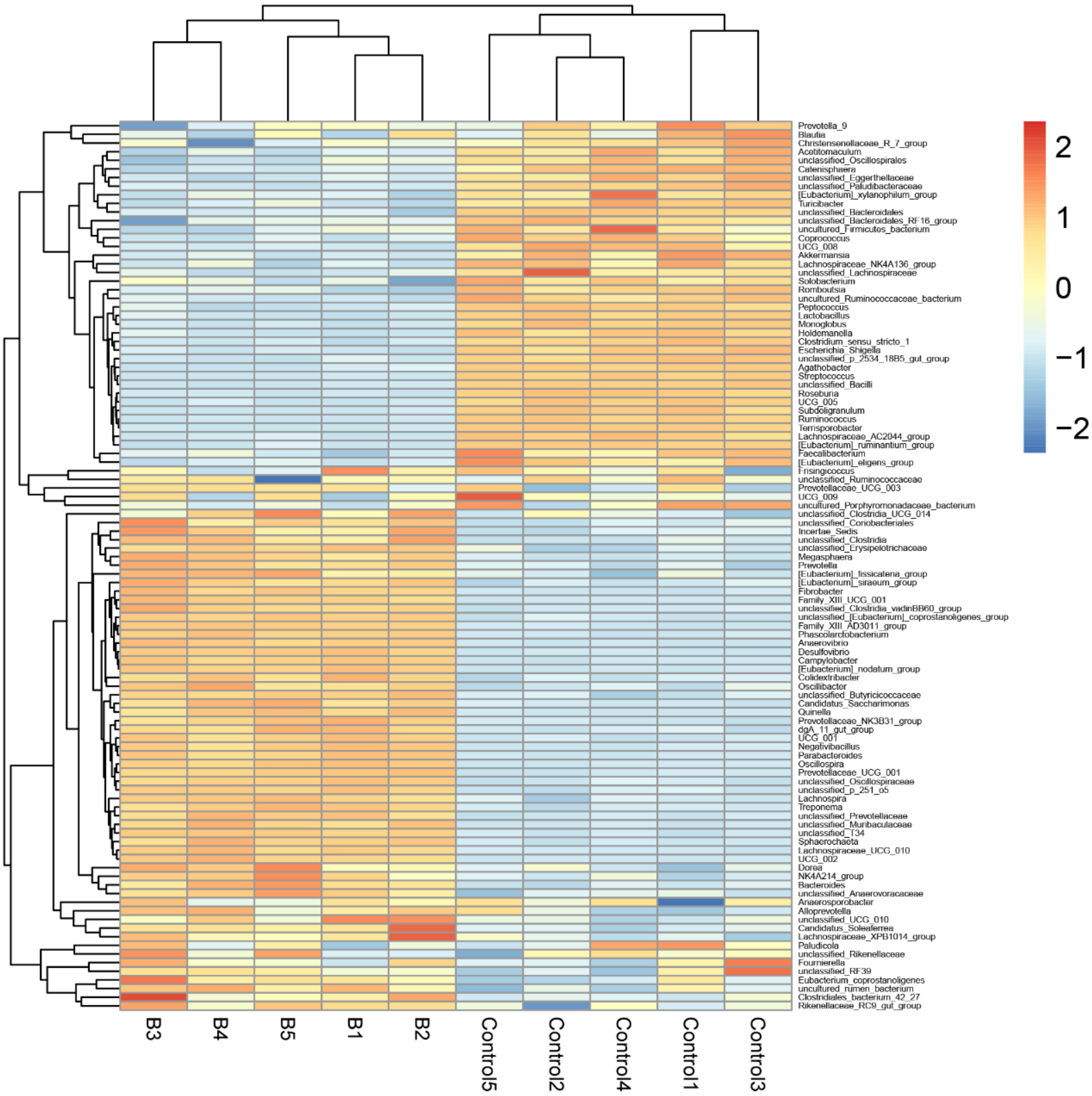

Figure S2. Heatmap profile showing the dominant genera in the colon microbiota. Pigs in the control group were fed basal diet, and the diet of group B was the basal diet with 2% FCHs.

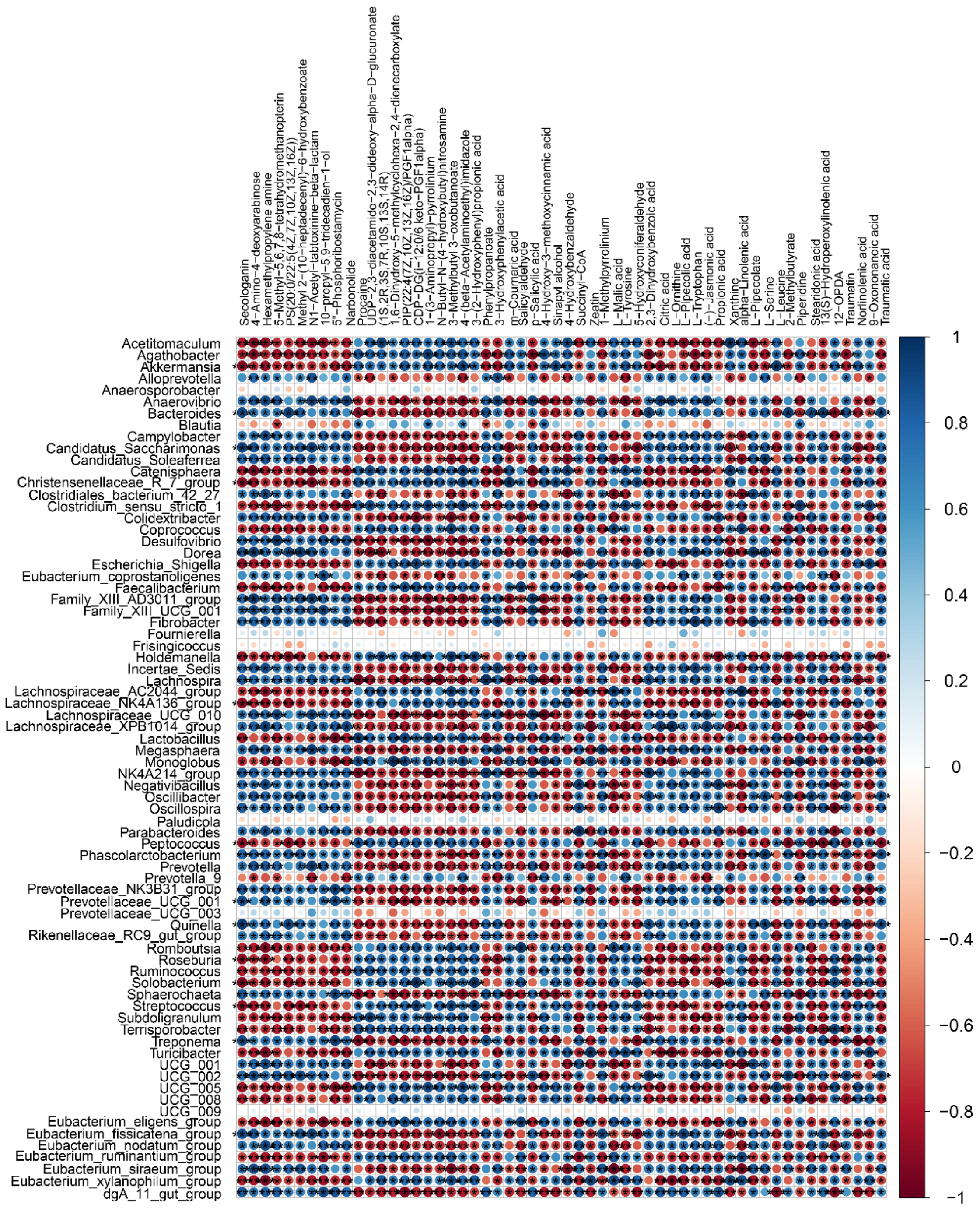

Figure S3. Spearmon correlation analysis results between dominant genera and major significantly different metabolites. * $p < 0.05$; ** $p < 0.01$; *** $p < 0.001$.

Table S1. Heatmap profile showing significantly different metabolites between the samples in groups B and control. Pigs in the control group were fed basal diet, and the diet of group B was the basal diet with 2% FCHs.
